# Protective Effects of Luteolin on Diabetic Nephropathy in STZ-Induced Diabetic Rats

**DOI:** 10.1155/2011/323171

**Published:** 2011-04-28

**Authors:** Guo Guang Wang, Xiao Hua Lu, Wei Li, Xue Zhao, Cui Zhang

**Affiliations:** ^1^Department of Pharmacology, Wannan Medical College, Wuhu 241002, China; ^2^Experimental Center for Function Subjects, Wannan Medical College, Wuhu 241002, China

## Abstract

Diabetic nephropathy is a long-term complication of diabetic mellitus. Many experimental evidences suggest that persistent hyperglycaemia generates intracellular reactive oxygen species (ROS) and upregulates transforming growth factor-b1 and extracellular matrix expression in mesangial and tubular epithelial cells, which is involved of free radicals in the pathogenesis of diabetes and more importantly in the development of diabetic complications. Antioxidants effectively inhibit high-glucose- and H2O2-induced transforming growth factor-b1 and fibronectin upregulation, thus providing evidence that ROS play an important role in high glucose-induced renal injury. The flavonoid luteolin has been shown to possess direct antioxidant activity, therefore we hypothesize that it may be useful in treatment of many chronic disease associated with oxidative stress, such as diabetic nephropathy via its antioxidant properties. Our results suggested that protection against development of diabetic nephropathy by luteolin treatment involved changes in superoxide dismutase (SOD) activity, the malondialdehyde (MDA) content and expression of Heme Oxygenase-1 (HO-1) protein.

## 1. Introduction

Herbal medicines are naturally occurring, plant-derived substances with minimal or no industrial processing that have been used to treat illness within local or regional healing practices. For a long time, herbal medicines or their extracts have been used to cure various diseases [1–3], because plant products are frequently considered to be less toxic and more free from side effects than synthetic ones [4]. Luteolin, a plant-derived flavonoid, has a variety of biological activities including well-known anti-inflammatory [5], antimutagenic, and antitumorigenic [6] properties. Moreover, it possesses direct antioxidant activity [7], which may be useful in treatment of many chronic disease associated with oxidative stress, such as cardiovascular diseases [8, 9], liver diseases [10, 11], diabetes [12], and aging [13]. Glucose uptake and utilisation by peripheral tissues, such as liver, muscle, and fat, is crucial for maintaining normal blood glucose level.

Many experimental evidences suggest the involvement of free radicals in the pathogenesis of diabetes [14] and more importantly in the development of diabetic complications [12, 15, 16]. Free radicals are capable of damaging cellular molecules, DNA, proteins, and lipids leading to altered cellular functions. Many recent studies reveal that antioxidants capable of neutralizing free radicals are effective in preventing experimentally induced diabetes in animal models [17, 18] as well as reducing the severity of diabetic complications [16].

Diabetic nephropathy is one of the most serious complications of diabetes and the most common cause of end-stage renal failure. At present, diabetic kidney disease affects about 15%–25% of type I diabetes patients [19] and 30%–40% of patients with type II diabetes [20]. Diabetic nephropathy is characterized by specific renal morphological and functional alterations. ROS play an important role in high glucose-induced renal injury [21, 22]. Flavonoids are abundant plant phenolic compounds. More than 6000 have been identified to date, and some have been shown to possess hypoglycemic and antidiabetic activities [23]. There is no report on luteolin relating to protective role on diabetic nephropathy. Therefore, the present study was aimed at determining the protective effects of luteolin on diabetic nephropathy in STZ-induced DM.

## 2. Methods

All experiments were performed in accordance with the Guide for the Care and Use of Laboratory Animals of the Chinese National Institutes of Health. Forty male Sprague-Dawley rats, weighing 180–220 g, were procured from an authorized firm in Nanjing, China. They were housed in a standard animal facility under controlled environmental conditions at room temperature 22 ± 2°C and 12-hour light-dark cycle and received a standard pellet diet and water *ad libitum*. At the beginning of the experimental period, rats were assigned randomly into four groups: the control group given a standard chow diet, the control group given a luteolin diet, the diabetic group given a standard chow diet, and the diabetic group given a luteolin diet. Type I diabetes was induced as described previously [24]. Briefly, diabetes mellitus was induced by a single intraperitoneal injection of streptozotocin (STZ, 70 mg/kg) dissolved in 0.1 M citrate buffer (pH 4.5) in male Sprague-Dawley rats. The control group rats were treated with the same volume citrate buffer. Diabetes was confirmed at 48 h after STZ injection by measuring the glucose concentrations of peripheral blood obtained from the tail vein (One Touch SureStep Meter, LifeScan, Calif, USA). All animals had free access to food and water. Diabetes was diagnosed by a sustained glucose concentration >15 mmol/L. After diabetes was confirmed, luteolin feeding was started at a dose of 200 mg/kg dissolved in distilled water using an intragastric tube. The rats were anaesthetized with pentobarbital sodium and sacrificed eight weeks after confirming high blood glucose level. Blood samples and kidney were collected for biochemical analyses. A 24-h urine was collected on the day before the blood sample and aliquots were taken.

### 2.1. Biochemical Analyses

Blood samples for the measurement of blood chemistry were drawn into prechilled EDTA-containing tubes and immediately placed on ice. Blood samples were centrifuged at 2300 g for separation of serums and stored at −70°C until assay. Serums were used for the estimation of glucose, 24-h urea protein, blood urea nitrogen (BUN), and creatinine as described previously [24, 25]. The levels of serum triacylglycerol (TG), total cholesterol (TC), high density lipoprotein (HDL), and low density lipoprotein (LDL) were measured as previously described [26]. Lipids in livers such as TC and TG were also estimated by the above-mentioned methods.

## 3. Antioxidant Measurement

Kidney homogenate (10%, w/v) was prepared with 0.1 M PBS and centrifuged at 12000 g for 10 min. The supernatant was used to determine superoxide dismutase (SOD) activity and Malondialdehyde (MDA) levels with commercially available kits (Nanjing Jiancheng Bioengineering Institute). 

### 3.1. Luteolin on Histology

At the end of followup, all rats were euthanized with high-dose pentobarbital. The kidneys were immediately removed and fixed in 4% neutral formalin for 2 hours. Three short-axis slices of renal tissue were obtained and embedded in paraffin. Sections were cut at 4 mm with a microtome and deparaffined with xylene. They were stained with Hematoxylin-Eosin (H-E) staining. Stained kidney sections were observed under a light microscope at magnifications of 400x.

### 3.2. Luteolin on Heme Oxygenase-1 and Phosphorylated Akt

Kidney samples (0.2 g) were lysed and homogenized in 2 mL of lysis buffer (10 mM Tris-buffered saline, 1 mM EDTA, 1 mM EGTA, 2 mM sodium orthovanadate, 0.2 mM PMSF, 2 *μ*g/mL leupeptin, 2 *μ*g/mL aprotinin, and 1% Triton X-100) for 30 min on ice and cleared by centrifugation at 13,000 g for 15 min at 4°C. Total protein concentration was determined in the supernatant using the Bradford assay (Bio-Rad Laboratories, Hercules, Calif, USA). For each lane, equal amounts of protein were mixed with sodium dodecyl sulfate (SDS) sample buffer and boiled for 5 minutes. Samples were separated on a 10% sodium dodecyl sulfatepolyacrylamide gel and then transferred to 0.2-*μ*m nitrocellulose membrane. Nitrocellulose blots were blocked by incubation in TBST (10 mM Tris-HCl, pH 7.5, 150 mM NaCl, and 0.1% Tween 20) containing 5% nonfat milk for 1 h at room temperature and then incubated with a rabbit polyclonal anti-Heme oxygenase-1 (HO-1), AKT/PKB, phospho-AKT/PKB, *β*-actin antibody (1 : 500 dilution) overnight at 4°C. After 3 washing steps, a secondary antirabbit antibody (1 : 10,000 dilution) was added and incubated for 1 hour. After rinsed with wash buffer for three times, the reaction was visualized by DAB.

#### 3.2.1. Statistical Analysis

All values are expressed as mean ± s.e.m. Statistical evaluation was done using one way analysis of variance (ANOVA) followed by Duncan's multiple range test (DMRT) by using SPSS 11.09 for windows. The significance level was set at *P* < .05.

## 4. Results

In this study, the STZ-treated diabetic rats developed uncontrolled type 1 diabetes mellitus. Rats that had received streptozotocin became diabetic at a frequency of 80%. All the rats had well-developed signs of diabetes after 2 weeks of STZ administration, that is, hyperglycaemia, glycosuria, polyuria, increased water consumption, and weight loss. Diabetes was associated with reduced body weight when compared with the control rats. Changes in initial and final body weight (BW) of normal control and experimental groups are shown in Table 1. As expected, marked BW loss was observed in diabetic rats. However, luteolin treatment appeared to protect the diabetic rats from massive BW loss. Luteolin-treated rats showed a recovery in final BW which was close to that of normal control rats.

### 4.1. Biochemical Finding

Blood glucose level was very high in DM group and DM luteolin group in 48 h after STZ injection. After 8 weeks, treatment with luteolin showed a significant fall of blood glucose level in diabetic rats (Table 2). BUN, serum creatinine, and 24-h urea protein were measured, and the results are shown in Table 3. The levels of BUN, 24 h urea protein, and creatinine were significantly increased in STZ-induced diabetic rats when compared with those of normal control rats treated and untreated with luteolin. Administration of luteolin at 200 mg/kg to diabetic rats tends to bring the values to near normal. The levels of blood urea nitrogen, 24 h urea protein, and creatinine were significantly decreased.

#### 4.1.1. Serum and Tissue Lipids Profile

Effects of treatment with luteolin to diabetic rats on serum lipids like TC, TG, serum high density lipoprotein (HDL), and low-density lipoprotein (LDL) are presented in Table 4. As was shown in Table 4 that the levels of serum TC, TG, and LDL were significantly increased in diabetic rats when compared with those of normal control rats, while the level of serum HDL was significantly decreased in diabetic rats when compared to that of normal control rats. The serum lipids like TC, TG, and LDL were significantly decreased and HDL was significantly increased in diabetic rats treated with luteolin when compared with those of diabetic rats.

Furthermore, there were significant increases of TC and TG in kidney in STZ-induced diabetic rats when compared to those of normal control rats. On the other hand, TC and TG levels in kidney were significantly decreased in diabetic rats treated with luteolin when compared to those of diabetic rats (Table 5).

### 4.2. Antioxidant Effects of Luteolin

**Figure 1:**
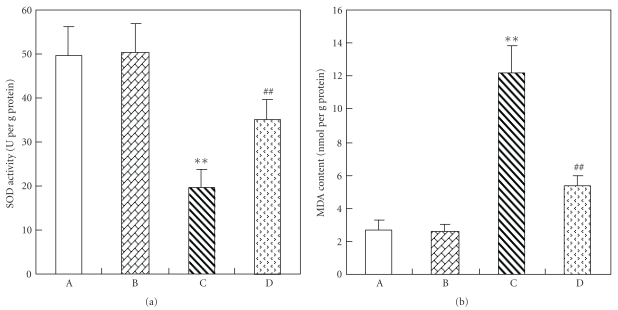
Changes of antioxidant effects in the kidney of each group. (A) Control rat. (B) Control rat with luteolin. (C) Diabetic rat. (D) Diabetic rats with luteolin. The kidney specimen of the diabetic group showed a markedly decrease of SOD activity and increase of MDA content (C). **P < .01, significantly different from control group and control luteolin group. ^##^ *P* < .01, significantly different from DM group.

As shown in Figure 1, SOD activity was significantly decreased and MDA level significantly increased in the kidney of STZ-induced diabetic rats when compared with those in normal control rats. Luteolin significantly enhanced SOD activity and reduced MDA level in kidney homogenates.

### 4.3. Histopathological Finding

**Figure 2:**
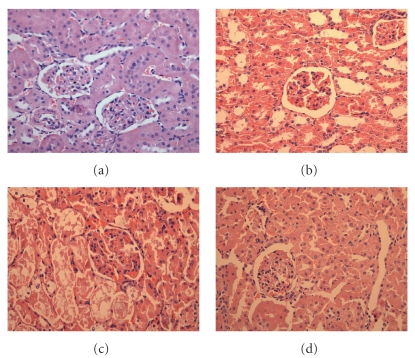
Photomicrographs of H-E staining in the kidney of each group. (a) Control rat. (b) Control rat with luteolin. (c) Diabetic rat. (d) Diabetic rats with luteolin. The kidney specimen of the diabetic group showed markedly severe destruction in glomerular and tubulointerstitial lesions such as glomerular sclerosis atrophy, interstitial expansion, and interstitial cellular infiltration (c). General morphology of glomerulus and tubulointerstitial lesions were much improved and showed quite normal appearance.

Diabetic nephropathy is characterized by an expansion of glomerular mesangium, which is caused by mesangial cell proliferation and excessive accumulation of ECM [27]. As demonstrated in Figure 2, in the normal rats, the outer cortical glomerulus was of normal size and configuration. Moreover, the mesangium contained the usual complement of cells and matrix without ECM accumulation. The glomeruli from diabetic rat kidneys were dramatically different in appearance.

**Western Blot**

**Figure 3:**
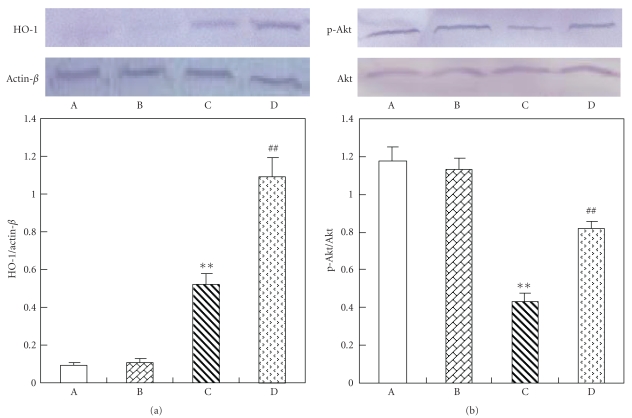
Western blot analysis in the kidney of each group. (A) Control rat. (B) Control rat with luteolin. (C) Diabetic rat. (D) Diabetic rats with luteolin. The kidney specimen of the diabetic group showed markedly a elevated expression of HO-1 and decreased p-Akt (C). **P < .01, significantly different from control group and control luteolin group. ^##^ *P* < .01, significantly different from DM group.

Our data show a significant increase expression of HO-1 protein in diabetic rat kidneys compared with that in the normal. Treatment with luteolin to diabetic rats increased expression of HO-1 protein (Figure 3). We also documented the fall of serine-473 phosphorylation of AKT/PKB in diabetic rat kidneys compared to that in the normal rat kidneys. Luteolin treatment increased AKT/PKB phosphorylation in diabetic rat kidneys compared with that in the untreated diabetic rat kidneys (Figure 3).

## 5. Discussion

In the presence study, we provide evidence that protection against the development of diabetic nephropathy by luteolin treatment involves changes in the expression of HO-1 and p-Akt. Our results show that luteolin treatment prevented the development of diabetic nephropathy by significantly lowering BUN and creatinine in diabetic animals. Luteolin-fed rats had less renal injury. This could be explained that there was increased clearance of blood urea and creatinine by the kidney or that there was decreased protein degradation. Moreover, luteolin also prevented the increase in 24-h urea protein in diabetic rats.

Increased polyol pathway activity induced by hyperglycaemia has been reported to contribute to abnormalities such as increased osmotic and oxidative stress factors that have been cited as promoters of diabetic microvascular diseases including diabetic nephropathy [28]. High plasma MDA level and decreased SOD activity are found in diabetes [29]. Antioxidants (e.g., vitamins C and E) protect against the development of diabetic nephropathy [30]. Various biological actions of luteolin are mediated by inhibiting oxidative stress [7] and inflammation [5]. Moreover, luteolin has been reported to mediate its effects by modulating several important molecular targets, including transcription factors (nuclear factor-kB and activating protein-1) [31], enzymes (inducible nitric oxide synthase inhibitor) [32], and cytokines (tumour necrosis factor-a, interleukin-1, interleukin-6, and chemokines) [33]. In present study, luteolin was found to decreased MDA level and increased SOD activity in kidney homogenate. 

The abnormal high concentration of serum lipids is mainly due to increase in the mobilization of free fatty acids from the peripheral fat deposits, because insulin inhibits the hormone sensitive lipase production. Therefore, the elevated level of serum lipids in DM causes the risk of diabetic nephropathy [34, 35]. It has been well established that DM alters the normal metabolism of tissues like kidney. The administration of luteolin to diabetic rats tends to bring the values to near normal. Luteolin is known to have antioxidant properties [7], and this may reduce the susceptibility of lipids to oxidation and stabilize the membrane lipids, thereby reducing oxidative stress. However, administering luteolin to diabetic rats tends to bring the values to near normal. Thus, luteolin treatments exhibited hypocholesterolaemic, hypotriglyceridaemic, and hypophospholipidaemic effects while at the same time increasing the HDL.

Increased plasma creatinine level and BUN are indications of the development of diabetic nephropathy in diabetic rats [36, 37]. BUN and creatinine levels are higher in rats with diabetic nephropathy than those in normal rats [38]. Maintenance of these biochemical variables closer to those in control rats by luteolin treatment suggests that luteolin plays a role, either directly or indirectly, in providing protection against diabetic nephropathy or delay in its development. 

Heme oxygenase-1 (HO-1), the inducible isoform of the HO system, is a rate-limiting enzyme which converts heme into equimolar amounts of iron, carbon monoxide, and biliverdin. HO-1 is thought to have antioxidant and cytoprotective roles [39]. The products of the HO reaction, biliverdin, and carbon monoxide can be toxic at very high concentrations. However, recent evidence indicates that they are not toxic at physiological concentrations in normal cells and that they may also have important antioxidant, anti-inflammatory, or anti-apoptotic properties [40, 41]. AKT protein is a serine/threonine kinase and a downstream effector of PI3-K. It plays a crucial role in a variety of cellular events such as apoptosis, cell cycle progression, and transcriptional regulation [42]. The recent studies indicated that the activated AKT account for the modulation of cardiac function [43], and HO-1 may be induced by inflammation and/or oxidative stress, which generates transcription factors activated by p-AKT, then increased the level of HO-1 protein [44, 45]. Diabetes was associated with oxidative stress; therefore, the level of HO-1 protein evelated. Our results show that luteolin increases the level of HO-1 protein. These results suggest that one of the mechanism of the renoprotective effect of luteolin may be related to increasing HO-1 expression and elevating antioxidant in diabetic nephropathy. 

In conclusion, the results from this study show that luteolin may prevent morphological destruction of kidney due to diabetes mellitus. Further studies are required to determine the exact mechanism of the renoprotective effect of luteolin.

## 6. Conclusions

From the above results, it may be concluded that luteolin may prevent the morphological destruction of the kidney that is associated with diabetes mellitus and may improve the redox balance in the kidney. Therefore, long-term control of antioxidant levels using a luteolin diet may prevent the progression of diabetes mellitus and prevent nephropathy.

## Figures and Tables

**Table 1:** Weight changes of the rats.

	Initial (g)	Final (g)	
Control group	190.1 ± 9.5	386.1 ± 25.7	*P* < .001
Control luteolin group	189.4 ± 10.2	383.9 ± 23.5	*P* < .001
DM group	195.9 ± 11.0	169.6 ± 12.8	*P* < .001
DM luteolin group	194.3 ± 13.0	201.3 ± 17.8	

**Table 2:** Change of Blood glucose.

	48 h (mmol/L)	8 weeks (mmol/L)	
Control group	5.06 ± 0.47	5.51 ± 0.82	
Control luteolin group	4.98 ± 0.56	5.31 ± 0.83	
DM group	22.25 ± 2.56	21.48 ± 2.28	
DM luteolin group	21.73 ± 2.59	13.66 ± 1.84	*P* < .01

**Table 3:** Biochemical analysis in blood.

	BUN (mmol/L)	Creatinine (mmol/L)	24-h urea protein (mg)
Control group	4.67 ± 0.55	45.06 ± 4.49	4.38 ± 0.94
Control luteolin group	4.51 ± 0.47	43.83 ± 4.76	4.26 ± 0.63
DM group	14.59 ± 0.76**	89.15 ± 6.26**	36.41 ± 4.51**
DM luteolin group	8.82 ± 0.80^##^	65.08 ± 5.32^##^	12.61 ± 2.54^##^

Values are expressed as mean ± SD of eight samples from each group. **P < .01, significantly different from control group and control luteolin group. ^##^ *P* < .01, significantly different from DM group.

**Table 4:** Change of serum lipids profile.

Groups	TC (mmol/L)	TG (mmol/L)	LDL (mmol/L)	HDL (mmol/L)
Control group	1.53 ± 0.29	0.71 ± 0.14	2.02 ± 0.43	1.35 ± 0.23
Control luteolin group	1.48 ± 0.24	0.69 ± 0.13	2.12 ± 0.40	1.31 ± 0.25
DM group	5.60 ± 0.71**	2.65 ± 0.51**	7.04 ± 0.85**	0.41 ± 0.13**
DM luteolin group	2.62 ± 0.35^##^	1.05 ± 0.14^##^	3.47 ± 0.58^##^	0.74 ± 0.17^##^

Values are expressed as mean ± SD of eight samples from each group. **P < .01, significantly different from control group and control luteolin group. ^##^ *P* < .01, significantly different from DM group.

**Table 5:** Change of kidney lipids profile.

	TC (mg per g protein)	TG (mg per g protein)
Control group	5.74 ± 0.37	4.51 ± 0.34
Control luteolin group	5.68 ± 0.35	4.39 ± 0.35
DM group	10.90 ± 0.78**	10.68 ± 0.75**
DM luteolin group	6.79 ± 0.69^##^	6.14 ± 0.48^##^

Values are expressed as mean ± SD of eight samples from each group. **P < .01, significantly different from control group and control luteolin group. ^##^ *P* < .01, significantly different from DM group.
